# Influence of Salinity Stress on Color Parameters, Leaf Pigmentation, Polyphenol and Flavonoid Contents, and Antioxidant Activity of *Amaranthus lividus* Leafy Vegetables

**DOI:** 10.3390/molecules27061821

**Published:** 2022-03-11

**Authors:** Md. Nazmul Hossain, Umakanta Sarker, Md. Sharif Raihan, Asma A. Al-Huqail, Manzer H. Siddiqui, Shinya Oba

**Affiliations:** 1 Department of Genetics and Plant Breeding, Faculty of Agriculture, Bangabandhu Sheikh Mujibur Rahman Agricultural University, Gazipur 1706, Bangladesh; nazmul99@gmail.com (M.N.H.); sharifdgpb@yahoo.com (M.S.R.); 2 Department of Botany and Microbiology, College of Science, King Saud University, Riyadh 11451, Saudi Arabia; aalhuqail@ksu.edu.sa (A.A.A.-H.); mhsiddiqui@ksu.edu.sa (M.H.S.); 3 Laboratory of Field Science, Faculty of Applied Biological Sciences, Gifu University, Yanagido 1-1, Gifu 501-1193, Japan; soba@gifu-u.ac.jp

**Keywords:** color parameters, antioxidant leaf pigmentation, polyphenols, flavonoids, antioxidant activity, salinity

## Abstract

This is the first attempt to evaluate the impact of four salinity levels on the color parameters, pigments, polyphenols, flavonoids, and antioxidant capacities of four promising *A. lividus* genotypes. The color parameters, such as the yellowness/blueness (b*) and the chroma (C*); the antioxidant components, such as the polyphenols and flavonoids; and the antioxidant capacities of the leaves were remarkably increased by 39, 1, 5, 10 and 43%, respectively, at 50 mM of NaCl, and by 55, 5, 60, 34, 58 and 82%, respectively, at 100 mM NaCl concentrations. The green tower and SA6 genotypes were identified as tolerant varieties. The total phenolic content (TPC) and the total flavonoid content (TFC) played vital roles in scavenging reactive oxygen species (ROS), and they would be beneficial for the human diet and would serve as good antioxidants for the prevention of aging, and they are also essential to human health. A correlation study revealed the strong antioxidant capacities of the pigments and antioxidant components that were studied. It was revealed that *A. lividus* could tolerate a certain level of salinity stress without compromising the antioxidant quality of the final product. Taken together, our results suggest that *A. lividus* could be a promising alternative crop for farmers, especially in saline-prone areas in the tropical and subtropical regions.

## 1. Introduction

Vegetable amaranth (*A. lividus*) is a C_4_ dicotyledonous plant that belongs to the family, Amaranthaceae. Vegetable amaranth is a rich source of nutrients, such as vitamin A, vitamin B (thiamine, riboflavin, niacin, and folate), vitamin C, calcium, magnesium, potassium, phosphorus, iron, zinc, copper, and manganese [1,2,3,4,5,6]. The leaves of amaranth are also enriched with different bioactive compounds, such as pigments, phenolics, and flavonoids [7,8,9,10]. Bioactive compounds play an essential role in protecting against cancer, atherosclerosis, arthritis, cataracts, emphysema, retinopathy, and neurodegenerative and cardiovascular diseases [11,12,13].

Because it is a major abiotic stress, salinity has serious adverse effects at different stages of plant development and growth [14]. The plant undergoes several morphological, physiological, anatomical, and biochemical changes during salinity stresses [15]. Salinity stress also leads to severe changes in photosynthesis and photorespiration activities [16]. At the initial stage of salinity stress, osmotic stress and ion toxicity occur, which may cause water loss, the interruption of the membrane, nutritional imbalance, changes in the enzyme and antioxidant activities, and an increase in the reactive oxygen species (ROS) [17]. An excessive accumulation of ROS (singlet oxygen, superoxide radical, hydroxyl radical, and hydrogen peroxide) can lead to oxidative damage in the plant [18]. In order to reduce the excessive ROS that is produced inside the cell, the plant accumulates many enzymatic and nonenzymatic antioxidants. The antioxidant enzymes include peroxidase (POX), superoxide dismutase (SOD), catalase (CAT), GSH reductase (GR), ascorbate peroxidase (APX), monodehydroascorbate dehydrogenase (MDHAR), and dehydroascorbate reductase (DHAR). In contrast, the nonenzymatic antioxidants include chlorophylls, carotenoids, polyphenols, flavonoids, tocopherols, Ascorbate (AsA), and glutathione (GSH) [19]. Betaxanthin and betacyanin also have shown antioxidant activities under stress conditions [20].

Amaranth is a leafy vegetable that is well acclimatized to different abiotic stresses [21,22,23,24] and that has multipurpose uses. The improvement of the natural antioxidants of this species in relation to the quantitative and qualitative aspects is dependent on different factors, such as the biological, environmental, biochemical, physiological, ecological, and evolutionary processes that are involved. Salinity stress can rapidly boost the antioxidant contents by altering this factor [25]. The betalains are absent in leafy vegetables, except for the Caryophyllales. The *A. lividus* leafy vegetable belongs to the order, Caryophyllales, and it attracts interest because of the presence of betalains (which are enriched with β-xanthins and β-cyanins) with excellent antioxidant activity.

Salt stress elevated the phenolics, flavonoids, antioxidant activity, and chlorophyll pigments in *Cichorium spinosum* [26]. Alam et al. [27] observed that different salinity treatment levels resulted in 8–35% increases in the TPC, about a 35% increase in the TFC, and 18–35% increases in the FRAP activity in purslane. Lim et al. [28] report that buckwheat that was treated with 10, 50, 100, and 200 mM NaCl concentrations resulted in increases in the phenolic compounds and carotenoids in the sprouts, compared to the control (0 mM). In the plant treated with 10, 50, and 100 mM of NaCl, after 7 days of cultivation, the phenolic contents of the sprouts were 57%, 121%, and 153%, respectively, which were higher than those of the control. Earlier, we tested *A. tricolor* genotypes under drought and salinity stress and found augmentations of the leaf color parameters, the antioxidant leaf pigments, the polyphenols, the flavonoid contents, and the antioxidant capacities. It is the first report of the effect of salinity stress on color parameters, antioxidant leaf pigmentation, polyphenols, flavonoids content, and antioxidant capacity of A. lividus leafy vegetable. Therefore, the present investigation aimed to study A. lividus genotypes in response to salinity stress in terms of color parameters, antioxidant leaf pigmentation, polyphenols, flavonoids content, and antioxidant activity 

## 2. Materials and Methods

### 2.1. Experimental Site

The experimental site is located at the center of the Madhupur tract (24.09° N latitude and 90.26° E longitudes), which is 8.4 m above sea level.

### 2.2. Plant Materials, Experiment Design, and Layout

Four vegetable amaranth (*A. lividus*) genotypes (i.e., green tower and red tower genotypes (Lal Teer-released varieties), and SA3 and SA6 genotypes (collected from the Department of Genetics and Plant Breeding, Bangabandhu Sheikh Mujibur Rahman Agricultural University)) were selected for the study. The experiment was conducted as a factorial design of the salinity treatments and varieties in a randomized complete block design (RCBD) with three replications.

### 2.3. Tray Preparation

The trays (38 cm × 24 cm × 10 cm) were filled with 5.5 kg of sandy loam soil. The upper portion of the tray (1–1.5 cm) was kept empty to hold the irrigation water. The fertilizers, N, P_2_O_5_, and K_2_O, were applied at 92, 48, and 60 kg ha^−1^, respectively. As the split dose, in the first installment of fertilizers, the N, P_2_O_5_, and K_2_O were used at 46, 48, and 60 kg ha^−1^, respectively, in the tray soil. In the second installment, the N, P_2_O_5_, and K_2_O were used at 46, 0, and 0 kg ha^−1^, respectively, seven days after sowing the seed (DAS).

### 2.4. Imposing Salinity Stress

The trays were well irrigated every day using fresh water, up to 9 DAS the seeds for the proper establishment and vigorous growth of the seedlings. The salinity stress treatment was started at 11 DAS, and it was continued up to 35 DAS (the edible stage). The salinity stress was applied by irrigating the respective trays with concentrations of 0 mM of NaCl (Control); 50 mM of NaCl, or medium salinity stress (MSS); 100 mM of NaCl, or severe salinity stress (SSS); and 150 mM of NaCl, or very severe salinity stress (VSSS), at 24 h intervals, from sowing to harvesting. The control plants received irrigation with normal water (without NaCl or freshwater). At 35 days after sowing (DAS), the leaves of *A. lividus* were harvested. All of the parameters were measured in three replicates.

### 2.5. Chemicals

The chemicals that were used in the study are acetone, methanol, ascorbic acid, the Folin–Ciocalteu reagent, sodium carbonate, aluminum chloride, potassium acetate, DPPH (2,2-diphenyl-picryl-hydrazyl), gallic acid, and quercetin.

### 2.6. Leaf Color Measurement

The color parameters (L*, a*, b*, and C*) were measured by a color meter (TES-135A, Plus, Taiwan) following Sarker and Oba [29,30,31]. The value of the L* indicates the lightness; a* indicates the degree of the red (+a*) or green (−a*) color; b* indicates the degree of the yellow (+b*) or blue (−b*) color; and the C* value, which expresses the chroma, indicates the leaf color intensity, which is calculated as: C* = (a^2^ + b^2^)^1/2^.

### 2.7. Determination of Chlorophyll and Total Carotene (μg·g^−1^)

Fresh leaves of each genotype were used for the chlorophyll *a*, chlorophyll *b,* chlorophyll *a*/*b,* total chlorophyll, and total carotene analyses, following the method prescribed by Sarker and Oba [32,33,34]. A single leaf disc of a 1 cm × 1 cm area was weighed and placed in a test tube. A total of 25 mL of 80% acetone was added, and the test tube was kept in the dark for 48 h. The supernatant was obtained, and the spectrophotometer (HITACHI: 200-20) readings were recorded at 663 n, 646 n, and 470 nm wavelengths. Then, the chlorophyll *a,* chlorophyll *b*, total chlorophyll, and total carotene were calculated by using the following formulas:

Chlorophyll *a* (μg·mL^−1^) = (12.21 × Absorbance at 663) − (2.81 × Absorbance at 646)

Chlorophyll *b* (μg·mL^−1^) = (20.13 × Absorbance at 646) − (5.03 × Absorbance at 663)

Total chlorophyll (μg·mL^−1^) = (17.32 × Absorbance at 663) + (7.18 × Absorbance at 646)

Total carotene (µg·mL^−1^) = (1000 × Absorbance at 470) − (3.27 × Chlorophyll *a* value) − (104 × Chlorophyll *b* value)/229

Finally, the chlorophylls were calculated as μg·g^−1^ of the fresh leaf weight, and the total carotene was calculated as μg of the total carotene per g of the fresh leaf weight.

### 2.8. Determination of Betacyanin and Betaxanthin (μg·g^−1^)

From each genotype, the fresh leaf was used for the betacyanin analysis, and their average values were obtained according to the method that is prescribed by Sarker and Oba [35,36]. A total of 0.1 g of leaf was processed in a mortar and pestle and homogenized. A total of 10 mL of aqueous methanol (80% methanol containing 50 μM ascorbic acid) was added. The solution was then shaken for 30 min and centrifuged at 14,000 rpm at 4 °C temperature for 10 min. The supernatant was obtained, and the spectrophotometer (HITACHI: 200-20) reading was recorded at 540 and 475 nm wavelengths for the betacyanin and betaxanthin, respectively. Finally, the betacyanin and betaxanthin were calculated by the following formulas:

Betacyanin (μg·g^−1^) = (Abs. at 540 nm × DF × MW)/ε × L × leaf weight (g)

(further explained as “A = Absorption at 540 nm, DF = Dilution factor (how much extraction), MW = Molecular weight of betacyanin (g mol^−1^), ε = Extinction coefficients of betacyanin (62 × 10^6^ cm^2^ mol^−1^), L = Path length is the 1 cm cuvette”); and Betaxanthin (μg·g^−1^) = [(A × DF × MW)/ε × L × leaf weight (g).

Here, A is the absorption at 475 nm; DF is the dilution factor (the extraction amount); MW is the molecular weight of the betaxanthin (g mol^−1^); ε is the extinction coefficients of the betaxanthin (48 × 10^6^ cm^2^ mol^−1^); and L is the path length, which is the 1 cm cuvette.

### 2.9. Sample Extractions for TPC, TFC, and TAC Analyses

For the total polyphenol content, the total flavonoid content, and the total antioxidant activity determinations, the fresh leaf was harvested to make the extraction. The leaves were harvested 35 days after sowing and were dried overnight. The samples were ground with a mortar and pestle for chemical analysis. A total of 0.25 g of leaf powder was dissolved in 10 mL of 90% methanol in a tightly capped bottle. Then, the bottle was placed in a water bath (Thomastant T- N22S, Thomas Kagaku Co., Ltd., Tokyo, Japan) with shaking. After 1 h, the extract was filtered for further analytical assays of the polyphenols, the flavonoid content, and the antioxidant activity.

### 2.10. Determination of the Total Polyphenol Content

The phenolic content was determined by the Folin–Ciocalteu reagent [37,38]. A total of 50 μL of the leaf extract solution was placed in a test tube. Then, 1 mL of the Folin–Ciocalteu reagent (which had been previously diluted with distilled water; reagent: water = 1:4) was added, and the content was meticulously mixed. A total of 1 mL of Na_2_CO_3_ (10%) was added after 3 min, and the mixture was allowed to stand for 1 h in the dark. The optical density was taken at 760 nm by using a spectrophotometer (HITACHI, Tokyo, Japan). The concentration of the total phenolic compounds in the leaf extracts was determined as the μg·g^−1^ of gallic acid equivalent by using an equation (Y = 0.009X + 0.019) that was obtained from a standard gallic acid graph and the following formula: C = (c × v)/m, where C is the total phenolic content; c is the concentration of the gallic acid; v is the volume of the extract; and m is the weight of the crude extract in g. The results are expressed as the μg·g^−1^ gallic acid equivalent of the dry weight (dw).

### 2.11. Determination of Total Flavonoid Content

The aluminum chloride colorimetric method was followed to estimate the total flavonoid content [39,40]. For this, 500 μL of leaf extract was placed in a test tube, followed by 0.1 mL of 1 M potassium acetate, 2.8 mL of distilled water, 0.1 mL of 10% aluminum chloride, and 1.5 mL of methanol. The absorbance was measured at 415 nm using a spectrophotometer (HITACHI, Tokyo, Japan), after allowing the mixture to stand for 30 min at room temperature. A standard curve (Y = 0.013X) was created that used quercetin as the standard compound. The results are expressed as the μg·g^−1^ quercetin equivalent of the dry weight (dw).

### 2.12. Determination of Total Antioxidant Activity (TAC)

The TAC was measured by the DPPH (2,2-diphenyl-picryl-hydrazyl) radical degradation method [41,42]. A total of 10 μL of leaf extract was placed in the test tubes, and a total of 4 mL of distilled water was added. A total of 1 ml of 250 μM of DPPH solution was added. After that, the test tubes were allowed to stand for 30 min in the dark. Then, the absorbance was obtained against a blank at 517 nm using a spectrophotometer (HITACHI: 200-20, Tokyo, Japan). The antioxidant activity was calculated as the percentage of the inhibition relative to the control by using the following equation:

Antioxidant% = (A _blank_ − A _sample_/A _blank_) × 100

where A _blank_ is the absorbance of the control reaction, and A _sample_ is the absorbance of the test compound in the sample.

### 2.13. Statistical Analysis

The recorded data were arranged according to a randomized complete block design, with three replications for the statistical analysis (the mean performance analysis). The crop performances were subjected to an analysis of variation (ANOVA) with Statistix 8 software [43,44,45]. The data were evaluated by Tukey’s W test (*p* ≤ 0.05) to identify the differences between the means.

## 3. Result and Discussion

The analysis of variance (ANOVA) provided significant variations among the treatments for all of the considered traits. A wide range of variations was also reported in the agronomic traits of maize [46,47,48], rice [49,50,51,52,53,54,55,56,57,58,59,60,61,62,63], okra [64,65,66], broccoli [67], and coconut [68,69].

### 3.1. Color Parameters

Significant variations in the leaf color attributes were observed among the four genotypes of *A. lividus* at different levels of salinity stress (Table 1). Among the varieties, the green tower showed the highest L* value (53.64), followed by the red tower (36.51), and the SA6 (35.02). In contrast, the SA3 exhibited the lowest L* value (26.26). In the case of the a* value, the highest value was found in the SA3 (9.44), followed by the SA6 (8.17), and the red tower (−12.95), and the lowest value was observed in the green tower (−23.55). Conversely, the highest b* value was found in the green tower (22.06), followed by the red tower (13.63), and the SA6 (3.59), and the lowest value was obtained in the SA3 (−2.52) (Table 1). Khanam and Oba [10] analyzed the leaf colors of two amaranth species (*A. tricolor* and *A. hypochondriacus*) and they observed significant differences between the cultivars. Khandaker et al. [70] also report a similar result in red amaranth (*A. tricolor*). The highest C* value was observed in the green tower (32.30), while the lowest value was noticed in the SA6 (9.25), followed by the SA3 (9.77). These results agree with the results of Sarker and Oba [23], who report pronounced variations of the L*, a*, b*, and C* values among different *A. tricolor* genotypes under salinity stress.

**Figure 1:**
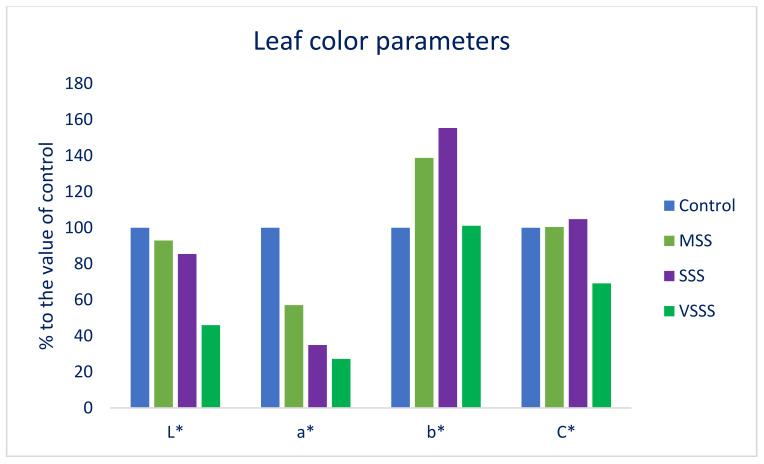
Changes in leaf color parameters (% to the value of control) under four salinity levels: Control (no saline water); MSS = medium salinity stress (50 mM NaCl); SSS = severe salinity stress (100 mM NaCl); and VSSS = very severe salinity stress (150 mM NaCl), in four selected *A. lividus* genotypes; L*, lightness; a*, redness/greenness; b*, yellowness/blueness; C* = chroma (leaf color intensity); (*n* = 5).

In terms of the salinity treatments, the highest L* value was obtained under the control treatment, followed by the MSS (43.38) and SSS (39.89) treatments, and the lowest value was obtained from the VSSS treatment (21.46). Increases in the salinity resulted in a significant reduction in the L* value from the control condition to the VSSS condition, following the order: Control > MSS > SSS > VSSS. In the case of the a* value, the highest value was found for VSSS (−2.35), followed by SSS (−3.01), and MSS (−4.92), and the lowest value was found in the control (−8.62). In contrast, the highest b* value was obtained under SSS (10.33), followed by MSS (10.31), and the control (8.64), and the lowest value was found under VSSS (7.51), as the red tower and SA3 genotypes failed to germinate under VSSS (Table 1). The highest C* value was obtained under SSS, followed by the control and MSS conditions, while the lowest value was recorded under VSSS. In the MSS, SSS, and VSSS conditions, the L* and a* values were decreased by 7 and 15%, 54 and 43%, and 65 and 73%, respectively (Figure 1). As the value of the a* is negative, the decrease in the a* value indicates the increment of redness, with the severity of the salinity stress ranging from MSS to VSSS. However, the b* and C* values were progressively increased by 39 and 1% under MSS, respectively, and by 55 and 5% under SSS, respectively, whereas the C* value was drastically reduced by 30% under VSSS conditions (Figure 1). Similarly, Sarker and Oba [23,24] also report the augmentation of the a* (+) redness, and the b* and C* values in *A. tricolor* genotypes with the increments of salinity stress. The extreme NaCl concentration under VSSS interrupted the physiology of *A. lividus*, which enhanced the drastic reductions in the L*, a*, b*, and C* values.

In the case of the interaction effect, the L*, a*, b*, and C* values ranged from 34.03 to 59.01, from 15.57 to 26.65, from −4.46 to 23.94, and from 8.82 to −34.48, respectively. The red tower genotype exhibited the highest L* value under the control condition, followed by the green tower under the control and MSS conditions. In contrast, the lowest L* value was reported in the SA6 genotype under the control condition. The highest a* value was found in the SA3 genotype under SSS conditions, followed by the SA3 genotype under MSS conditions, which indicates higher redness. In contrast, the lowest a* value was observed in the green tower genotype under the control condition, followed by the green tower genotype under MSS and SSS conditions, which indicates greenness. In the case of the b* value, the highest value was obtained from the green tower genotype under VSSS, followed by the green tower genotype under MSS and SSS conditions, which indicates yellowness. In contrast, under the control condition, the SA3 genotype exhibited the lowest b* value, which indicates blueness (Table 1). The leaf color of a product plays a crucial role in terms of the decisions, preferences, and acceptability of consumers. The antioxidant properties of a leafy vegetable depend on the color parameters of a leaf [71]. The genotypes that contain high pigments (anthocyanins, carotenoids, β-cyanin, β-xanthin, and betalain) have high redness and yellowness values. These results are fully in line with the results of Colonna et al. [71] and Sarker and Oba [23,24], who report the augmentations of the a* (+) redness, b*, and C* values in different leafy vegetables and *A. tricolor* genotypes, with the increments of light intensity and salinity stress, respectively.

### 3.2. Leaf Chlorophyll Content

Chlorophyll was found to be one of the most abundant pigments among the genotypes studied. The chlorophyll *a*, chlorophyll *b*, chlorophyll *a*/*b*, and the total chlorophyll content differed significantly among the four cultivars by a Tukey’s test at a 5% level of significance (Table 2). Among the varieties, the highest chlorophyll *a* content was obtained from the green tower genotype (355.07 μg·g^−1^), followed by the SA6 (346.14 μg·g^−1^) and SA3 (245.53 μg·g^−1^) genotypes. In contrast, the lowest chlorophyll *a* was found in the red tower genotype (221.99 μg·g^−1^). The SA6 genotype exhibited the highest chlorophyll *b* content (190.10 μg·g^−1^), followed by the green tower genotype (182.59 μg·g^−1^), whereas the lowest chlorophyll *b* content was recorded in the SA3 genotype (117.73 μg·g^−1^). The highest chlorophyll *a/b* value was exhibited in the SA3 genotype (2.09), which was followed by the green tower genotype (1.94) and the SA6 genotype (1.82), whereas the lowest chlorophyll *a/b* value was observed in the red tower genotype (1.77). In the case of the total chlorophyll, the highest total value was obtained by the green tower genotype (537.32 μg·g^−1^), followed by the SA6 genotype (535.80 μg·g^−1^), while the lowest total chlorophyll content was observed in the red tower genotype (347.22 μg·g^−1^) (Table 2). Sarker and Oba [11,24] and Ali et al. [72] report significant variations in the chlorophyll contents in different genotypes of *A. tricolor*.

**Figure 2:**
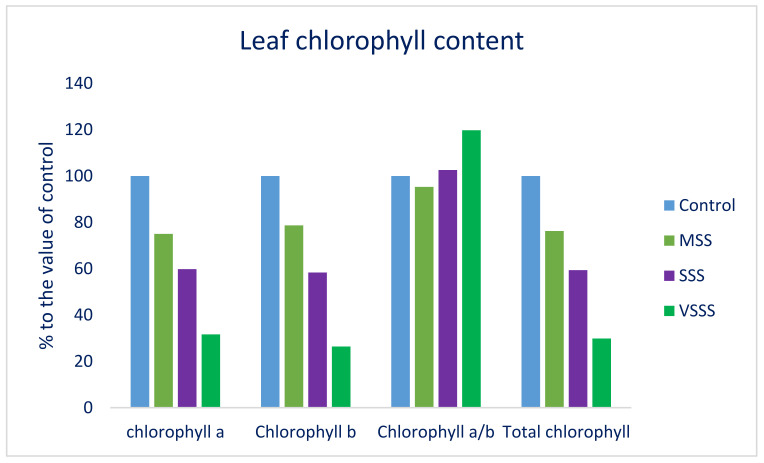
Impact on leaf chlorophyll content (% to the value of control) under four salinity levels: Control (no saline water); MSS = medium salinity stress (50 mM NaCl); SSS = severe salinity stress (100 mM NaCl); VSSS = very severe salinity stress (150 mM NaCl), in four selected *A. lividus* genotypes; (*n* = 5).

Within the salinity treatments, the highest chlorophyll *a* content was observed in the control treatment (438.65 μg·g^−1^), followed by the MSS (328.97 μg·g^−1^) and SSS (262.37 μg·g^−1^) treatments. In contrast, the VSSS (138.74 μg·g^−1^) condition showed the lowest chlorophyll *a* content. Similarly, the highest chlorophyll *b* content was observed in the control treatment (233.78 μg·g^−1^), followed by the MSS (183.96 μg·g^−1^) and SSS (136.33 μg·g^−1^) conditions, while the lowest chlorophyll *b* content was observed under VSSS (61.76 μg·g^−1^). The highest chlorophyll *a/b* value was observed under VSSS conditions (2.25), followed by the SSS (1.92) and MSS (136.33 μg·g^−1^) conditions, whereas the lowest chlorophyll *a/b* value was obtained under MSS conditions (1.79), followed by the control condition (1.88). In the case of the total chlorophyll, the highest total chlorophyll was observed in the control condition (672.29 μg·g^−1^), followed by the MSS (512.57 μg·g^−1^) SSS (398.92 μg·g^−1^) conditions, and the lowest total chlorophyll was obtained under VSSS conditions (200.57 μg·g^−1^). With the increase in the salinity, the chlorophyll *a,* chlorophyll *b,* and total chlorophyll contents were drastically and significantly reduced, from the control condition to the VSSS condition, following the order: Control > MSS > SSS > VSSS, while chlorophyll *a/b* was increased, following the order: MSS < Control < SSS < VSSS, which indicates that the severity of the salinity stress enhanced a drastic reduction in chlorophyll *b,* rather than in chlorophyll *a*. Under MSS, SSS, and VSSS conditions, the chlorophyll *a,* chlorophyll *b,* and the total chlorophyll content were decreased by 25, 40, and 68%, by 21, 42, and 78%, and by 24, 41, and 70%, respectively (Figure 2), whereas the chlorophyll *a/b* content was increased by 3 and 20% under SSS and VSSS conditions, respectively (Figure 2). Sarker and Oba [24] report a significant decrease in the chlorophyll content in *A. tricolor* with increasing salinity stress, and Jampeetong and Brix [73] also report a significant decrease in the chlorophyll content with increasing salinity in *Salvinia natans.*

In the variety × salinity stress interactions, the chlorophyll *a,* chlorophyll *b,* chlorophyll *a/b,* and the total chlorophyll ranged from 202.67 to 518.10 μg·g^−1^; from 95.06 to 323.91 μg·g^−1^; from 1.61 to 2.41 μg·g^−1^; and from 298.34 to 843.43 μg·g^−1^, respectively. The chlorophyll content decreased significantly with the increase in the salinity stress (Table 2). The highest chlorophyll *a* content was obtained from the SA6 genotype under the control conditions (518.10 μg·g^−1^), followed by the SA3 genotype under the control conditions (456.13 μg·g^−1^), and the green tower genotype under the control conditions (442.22 μg·g^−1^). In contrast, the lowest chlorophyll *a* content was observed in the SA3 genotype under SSS conditions (202.67 μg·g^−1^), followed by the SA6 under VSSS (238.29 μg·g^−1^), and the red tower under SSS (241.14 μg·g^−1^). The SA6 genotype showed the highest chlorophyll *b* content under the control condition (323.91 μg·g^−1^), followed by the green tower under the control condition (236.30 μg·g^−1^), and the green tower under the MSS condition (208.11 μg·g^−1^). In contrast, the lowest chlorophyll *b* content was observed in the SA3 genotype under SSS (95.06 μg·g^−1^), followed by the SA6 genotype under VSSS (111.01 μg·g^−1^), and the green tower genotype under VSSS (136.02 μg·g^−1^). The SA3 genotype showed the highest chlorophyll a/*b* content under the control condition (2.41), followed by the green tower under VSSS (236.30 μg·g^−1^). In contrast, the lowest chlorophyll a/*b* content was observed in the SA6 genotype under control conditions (1.61), followed by the red tower genotype under SSS (1.63), and the green tower genotype under MSS (1.65). The SA6 genotype exhibited the highest total chlorophyll content under the control condition (843.43 μg·g^−1^), which was followed by the green tower genotype under the control condition (676.03 μg·g^−1^), and the SA3 genotype under the control condition (646.21 μg·g^−1^). The SA3 genotype exhibited the lowest total chlorophyll content under SSS (298.34 μg·g^−1^) conditions, followed by the SA6 genotype under VSSS (348.86 μg·g^−1^) conditions, and the red tower genotype under SSS (388.14 μg·g^−1^) conditions (Table 2). The chlorophyll *a,* chlorophyll *b,* and total chlorophyll contents were dramatically reduced from the control condition to the VSSS condition, which indicates a drastic reduction in the chlorophyll *a,* chlorophyll *b,* and the total chlorophyll content with the severity of the salinity stress, while the chlorophyll *a/b* content was progressively increased from the control condition to the VSSS condition, with the increase in the salinity stress indicating a more drastic reduction in the chlorophyll *b* than in the chlorophyll *a*. Sarker and Oba [24] report a significant decrease in the chlorophyll content in *A. tricolor* with increasing salinity stress. Odjegba and Chukwunwike [74] report a substantial decline in the chlorophyll content with increasing salinity in *A. hybridus.* Salinity stress may reduce the leaf chlorophyll content through an increase in the chlorophyllase activity, which affects the membrane stability and weakens the protein–pigment–lipid complex [75]. Hanafy et al. [76] also report that this increase in chlorophyllase activities might be due to the absorption of excess ions, such as Mg and Fe, which were associated with the chloroplast formation.

### 3.3. Leaf Color Pigment Content

Pronounced and significant variations were observed for the betacyanin, betaxanthin, betalain, and the total carotene content across the four cultivars that were studied (*p* < 0.05). Among the varieties, the highest betacyanin content was reported in the SA6 genotype (128.20 ng·g^−1^), followed by the red tower (68.51 ng·g^−1^) and SA3 (68.39 ng·g^−1^) genotypes, whereas the betacyanin content was the lowest in the green tower genotype (64.83 ng·g^−1^) (Table 3). Ali et al. (2009) also observed variation in the betacyanin contents in different amaranth leaves. The highest betaxanthin and betalain contents were noted in the SA6 genotype (347.18 and 475.38 ng·g^−1^, respectively) which was followed by the SA3 genotype (219.10 and 287.49 ng·g^−1^, respectively), and the green tower genotype (204.54 and 269.37 ng·g^−1^, respectively). On the other hand, the lowest betaxanthin and betalain contents were reported in the red tower genotype (196.37 and 264.87 ng·g^−1^, respectively) (Table 3). In contrast, the highest total carotene content was noted in the green tower genotype (58.66 μg·g^−1^), followed by the SA3 (41.35 μg·g^−1^) and SA6 (41.27 μg·g^−1^) genotypes. The lowest total carotene content was noticed in the red tower genotype (27.15 μg·g^−1^). Khanam and Oba [10], Sarker et al. [7,9], and Sarker and Oba [12] also observed significant variations for the betacyanin, betaxanthin, betalain, and the total carotene content among *A. tricolor* cultivars.

**Figure 3:**
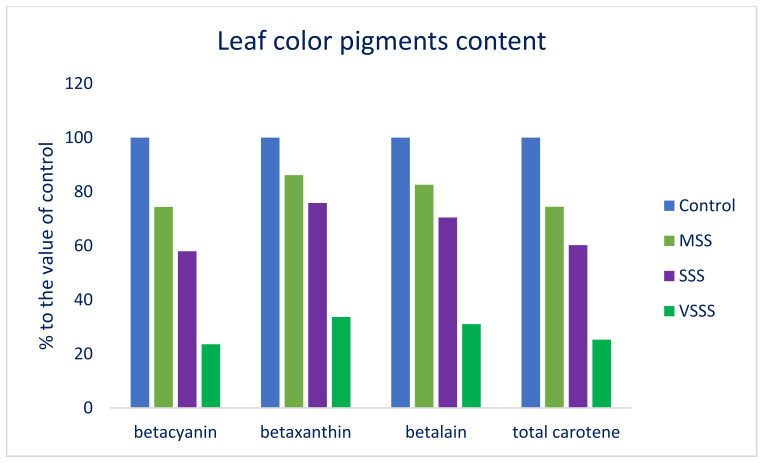
Response of leaf color pigment content (% to the value of control) under four salinity levels: Control (no saline water); MSS = medium salinity stress (50 mM NaCl); SSS = severe salinity stress (100 mM NaCl); VSSS = very severe salinity stress (150 mM NaCl) in four selected *A. lividus* genotypes; (*n* = 5).

Across the salinity treatments, the highest betacyanin, betaxanthin, betalain, and total carotene were observed in the control treatment (128.91, 327.21, and 456.12 ng·g^−1^, and 64.79 μg·g^−1^, respectively) followed by the MSS treatment (95.91, 281.87, and 377.78 ng·^−1^, and 48.23 μg·g^−1^, respectively), whereas the VSSS treatment (30.40, 110.02, and 140.41 ng·g^−1^, and 16.38 μg·g^−1^, respectively) exhibited the lowest betacyanin, betaxanthin, betalain, and total carotene contents. There was a drastic reduction in the betacyanin, betaxanthin, betalain, and total carotene with the increase in the salinity levels from the control condition to the VSSS condition in the following order: Control > MSS > SSS > VSSS (Table 3). Under MSS, SSS, and VSSS conditions, the betacyanin, betaxanthin, betalain, and total carotene were decreased by 26%, 42%, and 76%; 14%, 24%, and 66%; 17%, 30%, and 69%; and 26%, 40%, and 75%, respectively (Figure 3). Sehrawat et al. [77] also report a significant reduction in the carotene content with increasing salinity levels in mung beans.

Within the variety × salinity interaction, the betacyanin, betaxanthin, betalain, and total carotene content ranged from 38.74 to 159.76 ng·g^−1^; from 148.73 to 388.12 ng·g^−1^; from 187.47 to 547.87 ng·g^−1^; and from 20.64 to 70.88 μg·g^−1^, respectively. The SA6 genotype showed the highest betacyanin content under the control condition (159.76 ng·g^−1^), followed by the genotype SA6 under SSS (156.38 ng·g^−1^), and the red tower genotype under the control condition (136.77 ng·g^−1^). In contrast, the lowest betacyanin content was recorded in the green tower genotype under VSSS (38.74 ng·g^−1^), followed by the green tower genotype under SSS (40.87 ng·g^−1^), and the red tower genotype under SSS (63.83 ng·g^−1^) (Table 3). The SA6 genotype under the control condition (388.12 ng·g^−1^) exhibited the highest betaxanthin content, followed by the SA6 genotype under the MSS (368.26 ng·g^−1^) and SSS (340.99 ng·g^−1^) conditions. The green tower genotype showed the lowest betaxanthin content under VSSS (148.73 ng·g^−1^), followed by the green tower under SSS (163.53 ng·g^−1^) and MSS (214.80 ng·g^−1^) (Table 3). The SA6 genotype showed the highest betalain content under the control condition (547.87 ng·g^−1^), followed by the SA6 genotype under MSS (524.64 ng·g^−1^) and MSS (454.82 ng·g^−1^). Conversely, the green tower genotype exhibited the lowest betalain content under VSSS (187.47 ng·g^−1^), followed by the green tower under SSS (204.40 ng·g^−1^) and MSS (277.42 ng·g^−1^) (Table 3). The highest carotene content was obtained from the green tower genotype under the control condition (70.88 μg·g^−1^), followed by the SA3 under the control condition (70.80 μg·g^−1^), and the SA6 under MSS (66.20 μg·g^−1^). In contrast, the lowest carotene content was found in the red tower genotype under SSS (20.64 μg·g^−1^), followed by the red tower under MSS (24.80 μg·g^−1^), and the SA6 under VSSS (29.13 μg·g^−1^) (Table 3). Alam et al. [27] report that a reduction in the carotenoids with increasing salinity was also observed in *Portulaca oleracea*. Sehrawat et al. [77] show that, with the increasing salinity levels in mung beans, the carotene content was drastically reduced.

### 3.4. Total Polyphenols, Total Flavonoid Content, and Antioxidant Activity

The studied cultivars differed significantly for the total polyphenols, the total flavonoid content, and the antioxidant activity in response to the salinity stress (*p* < 0.05) (Table 4). Across the varieties, the highest total polyphenol content was obtained from the SA6 genotype (257.54 µg g^−1^ GAE dw), followed by the red tower genotype (207.68 µg g^−1^ GAE dw) and the SA3 genotype (193.36 µg g^−1^ GAE dw). In contrast, the green tower genotype (96.78 µg g^−1^ GAE dw) exhibited the lowest total polyphenol content among the varieties (Table 4). Gins et al. [51] report that the total polyphenol contents of amaranth cultivars varied remarkably among the cultivars. The highest total flavonoid content was documented in the SA6 genotype (107.36 µg g^−1^ QE dw), followed by the SA3 genotype (64.50 µg g^−1^ QE dw), and the red tower genotype (49.00 µg g^−1^ QE dw). In contrast, the lowest value was found in the green tower genotype (46.51 µg g^−1^ QE dw) (Table 4). Gins et al. [78] also report the variation in the total flavonoid contents in different amaranth cultivars. The SA6 genotype had the highest total antioxidant activity (16.71%), which was followed by the green tower (15.70%) and SA3 (12.43%) genotypes. In contrast, the red tower genotype showed the lowest total antioxidant activity (10.09%). Khandaker et al. [70] also found significant variations in red amaranths (*A. tricolor*). Moreover, Khandaker et al. [70] observed pronounced variations in the polyphenols, flavonoids, and antioxidant contents in *A. tricolor.*

**Figure 4:**
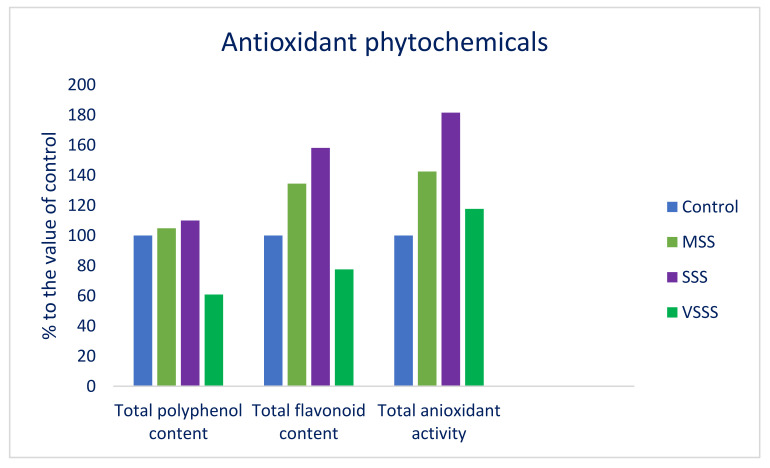
Variations in antioxidant phytochemicals (% to the value of control) under four salinity levels: Control (no saline water); MSS = medium salinity stress (50 mM NaCl); SSS = severe salinity stress (100 mM NaCl); VSSS = very severe salinity stress (150 mM NaCl), in four selected *A. lividus* genotypes; (*n* = 5).

Across the salinity treatments, the highest total polyphenol and total flavonoid contents were obtained under SSS (230.18 µg g^−1^ GAE dw and 89.95 µg g^−1^ QE dw, respectively), followed by MSS (219.44 µg g^−1^ GAE dw and 76.45 µg g^−1^ QE dw), while the lowest total polyphenol and total flavonoid contents were observed under VSSS (127.36 µg g^−1^ GAE dw and 44.09 µg g^−1^ QE dw, respectively) (Table 4). Increasing the salinity levels increased the total polyphenol and total flavonoid contents up to SSS conditions (100 mM of NaCl). The total polyphenol and total flavonoid contents were increased with the severity of the salt concentrations, from the control condition to the SSS condition, in the following order: Control < MSS < SSS (Table 4). Nevertheless, under VSSS, the total polyphenol and total flavonoid contents were drastically reduced compared to under the control condition. Under the MSS and SSS conditions, the polyphenol and flavonoid contents were progressively increased by 5 and 10%, and by 34 and 58%, respectively, whereas, under VSSS, the polyphenol and flavonoid contents were drastically reduced by 39 and 22%, respectively (Figure 4). Even though the green tower and SA6 genotypes survived because of their highly tolerant capacities, the SA3 and red tower genotypes failed to survive at very severe NaCl concentrations. Very severe salinity stress severely interrupted the physiological processes and growth of the plants, leaving them unable to cope with normal survival. As a result, the polyphenol and flavonoid contents were reduced under VSSS. The highest total antioxidant activity was recorded under SSS (18.84%), followed by MSS (14.79%), and VSSS (12.21%) (Table 4). In contrast, the lowest total antioxidant activity was observed in the control condition (10.38%). With the increase in the salinity levels, the total antioxidant activity was increased from the control condition to the VSSS condition, albeit the increments were sharper from the MSS condition to the SSS condition. The total antioxidant activity was sharply increased in the following order: Control < MSS < SSS < VSSS (Table 4). Under the MSS, SSS, and VSSS conditions, the total antioxidant activity increased by 43, 82, and 18%, respectively (Figure 4). Yarnia et al. [79] also demonstrate a similar increasing trend in the phenolic compounds in amaranth with increasing salinity stress. Moreover, Alam et al. [27] report a similar increasing trend in the flavonoid content in *Portulaca oleracea* under salinity stress. Salinity stress, which enhances the accumulation of the higher phenolic and flavonoid compounds within the plant cell, may accelerate the capacity of the plant to cope with oxidative stress under excessive salt concentrations or adverse conditions [80,81].

With regard to the interaction effects, the total polyphenol and total flavonoid contents, and the total antioxidant activity, ranged from 48.46 to 337.07 µg g^−1^ GAE dw; from 38.50 to 137.85 µg g^−1^ QE dw; and from 4.47 to 29.95%, respectively (Table 4). The SA6 genotype showed the highest total polyphenol content (337.07 µg g^−1^ GAE dw) under VSSS, followed by the red tower genotype under SSS (319.75 µg g^−1^ GAE dw), and the SA3 genotype under SSS (293.21 µg g^−1^ GAE dw). The lowest polyphenol content was found in the green tower genotype under the control condition (48.46 µg g^−1^ GAE dw). The highest total flavonoid content was detected in the SA6 genotype under VSSS (137.85 µg g^−1^ QE dw), followed by the SA3 genotype under SSS (122.69 µg g^−1^ QE dw), and the SA6 genotype under SSS (114.99 µg g^−1^ QE dw) (Table 4). In contrast, the lowest total flavonoid content was found in the green tower genotype under the control condition (38.50 µg g^−1^ QE dw), followed by the green tower genotype under MSS (44.76 µg g^−1^ QE dw) and SSS (50.28 µg g^−1^ QE dw) (Table 4). The highest total antioxidant activity was detected in the SA6 genotype under VSSS (29.953%), which was followed by the red tower genotype under SSS (22.28%), and the SA3 genotype under SSS (20.64%). In contrast, the SA6 genotype showed the lowest total antioxidant activity under the control condition (4.47%), which was followed by the red tower genotype under the control condition (8.56%), and the red tower genotype under MSS (9.51%) (Table 4). Sarker and Oba [22,23,24] reported an increase in the polyphenols and flavonoid contents and the antioxidant activity with the severity of the salt concentration in *A. tricolor*. They [24] also show that the salt-induced the increments of the polyphenol and flavonoid contents and the antioxidant activity mainly because of the increments of the phenolic acids and flavonoid compounds, which ultimately accelerated the total antioxidant activity in *A. tricolor*. The accumulation of higher phenolic and flavonoid compounds within the plant cell with the increase in the salinity stress may enhance the plant’s ability to reduce the harmful effects of salinity-induced oxidative stress [80,81].

### 3.5. Correlation Analysis

The chlorophyll exhibited highly significant correlations with chlorophyll *b*, the total chlorophyll, the total carotene content (TCC), the betacyanin, betaxanthin, and betalain, and the total polyphenol content (TPC). Chlorophyll *b* also showed a higher significant association with the total chlorophyll and the total carotene, and with the betacyanin, betaxanthin, and betalain contents (Table 5). The total chlorophyll demonstrated a significant correlation with the total carotene content (TCC), with the betacyanin, betaxanthin, and betalain, and with the total polyphenol content (TPC) (Table 5). This indicates that the increase in the color pigments was directly related to the increment in the TPC. The total carotene content (TCC) also showed a significant correlation with betacyanin, betaxanthin, and betalain. The betacyanin displayed higher interrelationships with the betaxanthin and betalain, and significant correlations with the total polyphenol content (TPC) and the total flavonoid content (TFC). The betaxanthin had significant associations with the betalain, the total polyphenol content (TPC), the total flavonoid content (TFC), and the total antioxidant activity (TAA). The betalain exhibited a significant interrelation with the total polyphenol content (TPC), the total flavonoid content (TFC), and the total antioxidant activity (TAA), which indicates that the betalamic pigments played a significant role in the antioxidant activity of *A. tricolor*. The total polyphenol content (TPC) demonstrated significant correlations with the total flavonoid content (TFC) and the total antioxidant activity (TAA).

A significant association between the total flavonoid content (TFC) and the total antioxidant activity (TAA) was observed (Table 5). This indicates that the TPC and the TFC played vital roles in the antioxidant activity of *A. tricolor*. Sarker and Oba [22,23,24] also noticed a strong association among the color pigments, polyphenols, flavonoids, and antioxidant activity of *A. tricolor* under salinity stress. Moreover, Khandaker et al. [70] observed a strong correlation between the accumulation of phenolic compounds and the antioxidant activity in amaranth.

## 4. Conclusions

Salinity stress significantly enhanced the b*, the chroma, chlorophyll *a/b*, the total polyphenol and flavonoid contents, and the total antioxidant activity of the leaf of an *A. lividus* leafy vegetable. Among the four varieties, the green tower and SA6 genotypes may be considered to be the best salinity-stress-tolerant varieties, as they survived up to very severe salinity stress (150 mM NaCl). The red tower and SA3 genotypes also showed salinity tolerance and survived up to extreme salinity stress. Under MSS and SSS conditions, the leaf color parameters, such as the b*, the c*, the total polyphenol and flavonoid contents, and the total antioxidant activity of *A. lividus* leaves were very high in comparison to the control conditions, which allows for the assignment of *A. lividus* as a valuable food source for human diets and health benefits. The interrelationships of salt-stressed amaranth reveal the potent antioxidant activity for the leaf pigments, the TPC, and the TFC. The leaf pigments, the TPC, and the TFC played vital roles in scavenging ROS, and they would be beneficial for human nutrition by serving as a good antioxidant and antiaging source for human health benefits. Furthermore, *A. lividus,* which is cultivated under salinity stress, could contribute to the high quality of the final product in terms of polyphenols, flavonoids, and antioxidants. It could be a promising alternative crop for farmers, especially in saline-prone areas and in the coastal belts of the world, and it could be recommended to the farmers of salt-affected areas.

## Figures and Tables

**Table 1:** Influence of salinity stress on leaf color parameters in four selected genotypes of *A. lividus* leafy vegetable.

Treatment	L*	a*	b*	C*
**Variety × Salinity stress**				
Green tower × Control	57.7 ± 0.6 b	−26.65 ± 0.4 n	17.80 ± 0.4 e	32.05 ± 0.8 c
Green tower × MSS	57.19 ± 0.4 c	−25.68 ± 0.3 m	23.01 ± 0.5 c	34.48 ± 0.7 a
Green tower × SSS	50.78 ± 0.3 d	−23.16 ± 0.3 l	23.7 ± 0.3 b	33.14 ± 0.6 b
Green tower × VSSS	48.87 ± 0.5 e	−18.717 ± 0.4 j	23.94 ± 0.4 a	30.38 ± 0.8 d
SA3 × Control	36.04 ± 0.3 i	8.39 ± 0.5 e	−4.46 ± 0.3 m	9.50 ± 0.7 j
SA3 × MSS	34.59 ± 0.04 j	13.79 ± 0.4 b	−3.22 ± 0.4 l	14.16 ± 0.8 h
SA3 × SSS	34.41 ± 0.4 j	15.57 ± 0.4 a	−2.41 ± 0.5 k	15.76 ± 0.7 g
SA3 × VSSS	NS	NS	NS	NS
Red tower × Control	59.01 ± 0.5 a	−22.66 ± 0.5 k	20.70 ± 0.3 d	30.69 ± 0.6 d
Red tower × MSS	47.39 ± 0.5 f	−15.88 ± 0.4 i	17.98 ± 0.4 e	23.99 ± 0.7 e
Red tower × SSS	39.65 ± 0.4 g	−13.25 ± 0.5 h	15.84 ± 0.4 f	20.65 ± 0.8 f
Red tower × VSSS	NS	NS	NS	NS
SA6 × Control	34.03 ± 0.5 k	6.45 ± 0.4 g	3.53 ± 0.4 j	8.82 ± 0.6 k
SA6 × MSS	34.37 ± 0.3 j	8.10 ± 0.3 f	3.49 ± 0.3 i	9.76 ± 0.7 j
SA6 × SSS	34.71 ± 0.2 j	8.81 ± 0.5 d	4.21 ± 0.5 h	11.16 ± 0.8 i
SA6 × VSSS	36.97 ± 0.4 h	9.33 ± 0.3 c	6.12 ± 0.4 g	9.89 ± 0.6 j
**Variety**				
Green tower	53.64 ± 0.4 a	−23.55 ± 0.4 d	22.11 ± 0.5 a	32.30 ± 0.6 a
SA3	26.26 ± 0.5 d	9.44 ± 0.3 a	−2.52 ± 0.3 d	9.77 ± 0.8 c
Red tower	36.51± 0.3 b	−12.95 ± 0.5 c	13.63 ± 0.4 b	18.80 ± 0.7 b
SA6	35.02 ± 0.4 c	8.17± 0.4 b	3.59 ± 0.3 c	9.25 ± 0.8 d
**Salinity stress**				
Control	46.70 ± 0.3 a	−8.62 ± 0.3 d	8.64 ± 0.5 b	11.38 ± 0.6 b
MSS	43.38 ± 0.4 b	−4.92 ± 0.4 c	10.31 ± 0.4 a	11.42 ± 0.7 b
SSS	39.89 ± 0.4 c	−3.01 ± 0.5 b	10.33 ± 0.3 a	11.93 ± 0.8 a
VSSS	21.46 ± 0.3 d	−2.35 ± 0.3 a	7.51 ± 0.4 c	7.87 ± 0.7 c
**Significance**				
Variety	*	*	*	*
Salinity stress	*	*	*	*
Variety × Salinity stress	*	*	*	*

L*, lightness; a*, (+) redness/(−) greenness; b*, (+) yellowness/(−) blueness; C*, chroma (leaf color intensity). Control (no saline water); MSS = medium salinity stress (50 mM NaCl); SSS = severe salinity stress (100 mM NaCl); VSSS = very severe salinity stress (150 mM NaCl); NS = no survival; mean values with different letters in the same columns are significantly different at *p* ≤ 0.05, (*, 5% level of significance), (*n* = 5).

**Table 2:** Impacts of salinity stress on leaf chlorophyll contents in four selected genotypes of *A. lividus* leafy vegetable.

Treatment	Chlorophyll *a* (μg·g^−1^)	Chlorophyll *b* (μg·g^−1^)	Chlorophyll *a*/*b*	Total Chlorophyll (μg·g^−1^)
**Variety × Salinity stress**				
Green tower × Control	442.22 ± 0.16 c	236.30 ± 0.13 b	1.87 ± 0.02 e	676.03 ± 0.33 b
Green tower × MSS	342.95 ± 0.15 d	208.11 ± 0.16 c	1.65 ± 0.04 i	548.65 ± 0.37 d
Green tower × SSS	318.42 ± 0.12 g	149.93 ± 0. 12 i	2.12 ± 0.03 c	471.18 ± 0.38 h
Green tower × VSSS	316.68 ± 0.13 g	136.02 ± 0.14 j	2.33 ± 0.03 b	453.41 ± 0.35 i
SA3 × Control	456.13 ± 0.12 b	189.36 ± 0.14 d	2.41 ± 0.04 a	646.21 ± 0.35 c
SA3 × MSS	323.31 ± 0.14 f	186.51 ± 0.13 e	1.73 ± 0.03 h	511.47 ± 0.34 f
SA3 × SSS	202.67 ± 0.13 l	95.06 ± 0.12 l	2.13 ± 0.02 c	298.34 ± 0.36 m
SA3 × VSSS	NS	NS	NS	NS
Red tower × Control	338.15 ± 0.16 e	185.54 ± 0.11 e	1.82 ± 0.04 f	523.47 ± 0.36 e
Red tower × MSS	308.67 ± 0.13 h	168.35 ± 0.13 g	1.83 ± 0.02 f	477.28 ± 0.34 g
Red tower × SSS	241.14 ± 0.15 j	147.71 ± 14 i	1.63 ± 0.03 i,j	388.14 ± 0.35 k
Red tower × VSSS	NS	NS	NS	NS
SA6 × Control	518.10 ± 0.12 a	323.91 ± 0.15 a	1.61 ± 0.02 k	843.43 ± 0.34 a
SA6 × MSS	340.92 ± 0.12 d	172.86 ± 0.17 f	1.97 ± 0.03 d	512.90 ± 0.34 f
SA6 × SSS	287.23 ± 0.16 i	152.62 ± 0.12 h	1.88 ± 0.03 e	438.01 ± 0.33 j
SA6 × VSSS	238.29 ± 0.15 k	111.01 ± 0.15 k	2.15 ± 0.02 c	348.86 ± 0.36 l
**Variety**				
Green tower	355.07 ± 0.14 a	182.59 ± 0.13 b	1.94 ± 0.04 b	537.32 ± 0.32 a
SA3	245.53 ± 0.13 c	117.73 ± 0.14 d	2.09 ± 0.02 a	364.00 ± 0.31 c
Red tower	221.99 ± 0.14 d	125.40 ± 0.14 c	1.77 ± 0.03 d	347.22 ± 0.38 d
SA6	346.14 ± 0.17 b	190.10 ± 0.14 a	1.82 ± 0.05 c	535.80 ± 0.33 b
**Salinity stress**				
Control	438.65 ± 0.16 a	233.78 ± 0.11 a	1.88 ± 0.02 c	672.29 ± 0.37 a
MSS	328.97 ± 0.13 b	183.96 ± 0.15 b	1.79 ± 0.02 d	512.57 ± 0.38 b
SSS	262.37 ± 0.14 c	136.33 ± 0.17 c	1.92 ± 0.03 b	398.92 ± 0.34 c
VSSS	138.74 ± 0. 14 d	61.76 ± 0.16 d	2.25 ± 0.04 a	200.57 ± 0.36 d
**Significance**				
Variety	*	*	*	*
Salinity stress	*	*	*	*
Variety × Salinity stress	*	*	*	*

Control (no saline water); MSS = medium salinity stress (50 mM NaCl); SSS = severe salinity stress (100 mM NaCl); VSSS = very severe salinity stress (150 mM NaCl); NS = no survival; different letters in column differed significantly by Tukey’s W test (*, 5% level of significance), (*n* = 5).

**Table 3:** Effects of salinity stress on leaf betacyanin, betaxanthin, betalain, and total carotene contents in four selected genotypes of *A. lividus* leafy vegetable.

Treatment	Betacyanin (ng·g^−1^)	Betaxanthin (ng·g^−1^)	Betalain (ng·g^−1^)	Total Carotene(μg·g^−1^)
**Variety × Salinity stress**				
Green tower × Control	117.09 ± 0.65 d	291.10 ± 0.42 g	407.63 ± 0.53 f	70.88 ± 0.04 a
Green tower × MSS	62.62 ± 0.63 k	214.80 ± 0.47 k	277.67 ± 0.51 l	66.20 ± 0.03 b
Green tower × SSS	40.87 ± 0.65 l	163.53 ± 0.43 l	205.35 ± 0.52 m	61.30 ± 0.03 c
Green tower × VSSS	38.74 ± 0.68 m	148.73 ± 0.39 m	188.25 ± 0.57 n	36.24 ± 0.04 g
SA3 × Control	102.01 ± 0.67 f	317.73 ± 0.45 d	418.82 ± 0.58 e	70.80 ± 0.05 a
SA3 × MSS	91.22 ± 0.64 g	293.18 ± 0.41 f	385.16 ± 0.55 g	56.44 ± 0.05 d
SA3 × SSS	80.31 ± 0.61 i	265.50 ± 0.47 h	347.01 ± 0.59 i	37.97 ± 0.04 g
SA3 × VSSS	NS	NS	NS	NS
Red tower × Control	136.77 ± 0.62 c	311.89 ± 0.40 e	446.98 ± 0.56 d	63.15 ± 0.05 c
Red tower × MSS	73.43 ± 0.62 j	251.23 ± 0.42 i	325.73 ± 0.52 j	24.80 ± 0.03 i
Red tower × SSS	63.83 ± 0.64 k	222.36 ± 0.46 j	288.21 ± 0.61 k	20.64 ± 0.04 j
Red tower × VSSS	NS	NS	NS	NS
SA6 × Control	159.76 ± 0.63 a	388.12 ± 0.49 a	547.49 ± 0.57 a	54.34 ± 0.03 e
SA6 × MSS	156.38 ± 0.68 b	368.26 ± 0.48 b	522.94 ± 0.64 b	45.46 ± 0.06 f
SA6 × SSS	113.83 ± 0.64 e	340.99 ± 0.47 c	455.11 ± 0.56 c	36.12 ± 0.04 g
SA6 × VSSS	82.843 ± 0.60 h	291.35 ± 0.42 g	373.25 ± 0.59 h	29.13 ± 0.06 h
**Variety**				
Green tower	64.83 ± 0.67 c	204.54 ± 0.48 c	268.28 ± 0.53 c	58.66 ± 0.06 a
SA3	68.39 ± 0.65 b	219.10 ± 0.41 b	288.53 ± 0.59 b	41.35 ± 0.07 b
Red tower	68.51 ± 0.63 b	196.37 ± 0.44 d	263.75 ± 0.54 d	27.15 ± 0.05 c
SA6	128.20 ± 0.67 a	347.18 ± 0.45 a	473.86 ± 0.51 a	41.27 ± 0.03 b
**Salinity stress**				
Control	128.91 ± 0.69 a	327.21 ± 0.42 a	458.64 ± 0.55 a	64.79 ± 0.05 a
MSS	95.91 ± 0.63 b	281.87 ± 0.47 b	378.71 ± 0.59 b	48.23 ± 0.04 b
SSS	74.71 ± 0.61 c	248.10 ± 0.45 c	323.15 ± 0.61 c	39.01 ± 0.03 c
VSSS	30.40 ± 0.61 d	110.02 ± 0.47 d	142.11 ± 0.59 d	16.38 ± 0.04 d
**Significance**				
Variety	*	*	*	*
Salinity stress	*	*	*	*
Variety × Salinity stress	*	*	*	*

Control (no saline water); MSS = medium salinity stress (50 mM NaCl); SSS = severe salinity stress (100 mM NaCl); VSSS = very severe salinity stress (150 mM NaCl); NS = no survival; the different letters in a column differed significantly by Tukey’s W test (*, 5% level of significance), (*n* = 5).

**Table 4:** Impact of salinity stress on total polyphenol content, total flavonoid content, and total antioxidant activity of four selected genotypes of *A. lividus* leafy vegetable.

Treatment	Total Polyphenol Content (µg g^−1^ GAE dw)	Total Flavonoid Content (µg g^−1^ QE dw)	Total Antioxidant Activity (%)
**Variety × Salinity stress**			
Green tower × Control	48.46 ± 0.05 n	38.50 ± 0.23 m	13.74 ± 0.16 f
Green tower × MSS	53.76 ± 0.06 m	44.76 ± 0.27 l	13.84 ± 0.15 f
Green tower × SSS	112.53 ± 0.07 l	50.28 ± 0.21 k	16.34 ± 0.12 e
Green tower × VSSS	172.38 ± 0.08 k	52.52 ± 0.28 j	18.88 ± 0.11 d
SA3 × Control	221.66 ± 0.07 i	53.35 ± 0.25 i,j	9.61 ± 0.17 g
SA3 × MSS	258.57 ± 0.06 e	81.96 ± 0.23 e	19.48 ± 0.10 d
SA3 × SSS	293.21 ± 0.07 c	122.69 ± 0.24 b	20.64 ± 0.14 c
SA3 × VSSS	NS	NS	NS
Red tower × Control	239.89 ± 0.05 g	54.88 ± 0.21 i	8.56 ± 0.13 h
Red tower × MSS	271.08 ± 0.06 d	63.76 ± 0.20 h	9.51 ± 0.16 g
Red tower × SSS	319.75 ± 0.06 b	77.36 ± 0.27 f	22.28 ± 0.14 b
Red tower × VSSS	NS	NS	NS
SA6 × Control	203.52 ± 0.05 j	66.80 ± 0.22 g	4.47 ± 0.12 i
SA6 × MSS	235.58 ± 0.08 h	109.81 ± 0.26 d	13.81 ± 0.15 f
SA6 × SSS	253.99 ± 0.05 f	114.99 ± 0.26 c	18.61 ± 0.13 d
SA6 × VSSS	337.07 ± 0.05 a	137.85 ± 0.29 a	29.95 ± 0.12 a
**Variety**			
Green tower	96.78 ± 0.06 d	46.51 ± 0.23 d	15.70 ± 0.12 b
SA3	193.36 ± 0.06 c	64.50 ± 0.26 b	12.43 ± 0.13 c
Red tower	207.68 ± 0.04 b	49.00 ± 0.25 c	10.09 ± 0.12 d
SA6	257.54 ± 0.05 a	107.36 ± 0.29 a	16.71 ± 0.16 a
**Salinity stress**			
Control	209.36 ± 0.04 c	56.89 ± 0.27 c	10.38 ± 0.11 d
MSS	219.44 ± 0.03 b	76.45 ± 0.33 b	14.79 ± 0.15 b
SSS	230.18 ± 0.03 a	89.95 ± 0.28 a	18.84 ± 0.15 a
VSSS	127.36 ± 0.03 d	44.09 ± 0.27 d	12.21 ± 0.12 c
**Significance**			
Variety	*	*	*
Salinity stress	*	*	*
Variety × Salinity stress	*	*	*

Control (no saline water); MSS = medium salinity stress (50 mM NaCl); SSS = severe salinity stress (100 mM NaCl); VSSS = very severe salinity stress (150 mM NaCl); NS = no survival; different letters in a column differed significantly by Tukey’s W test (*, 5% level of significance), (*n* = 5).

**Table 5:** Correlation coefficients for chlorophylls, total carotene contents, antioxidant leaf pigments, and TPCs, TFCs, and TAAs of four selected genotypes of *A. lividus* leafy vegetable under salinity stress.

Parameters	Chl *a* (μg·g^−1)^	Chl *b* (μg·g^−1)^	T Chl (μg·g^−1^)	TCC (μg·g^−1^)	Betacyanin (ng·g^−1^)	Betaxanthin (ng·g^−1^)	Betalain (ng·g^−1^)	TPC (µg g^−1^ GAE dw)	TFC (µg g^−1^ QE dw)	TAA (%)
Chl a		0.956 **	0.994 **	0.856 **	0.768 **	0.807 **	0.805 **	0.309 *	0.261	0.225
Chl b			0.982 **	0.784 **	0.784 **	0.794 **	0.800 **	0.270	0.212	0.170
T Chl				0.838 **	0.781 **	0.810 **	0.810 **	0.299 *	0.246	0.206
TCC					0.617 **	0.645 **	0.644 **	0.094	0.174	0.237
Betacyanin						0.948 **	0.974 **	0.495 **	0.499 **	0.209
Betaxanthin							0.995 **	0.662 **	0.650 **	0.429 **
Betalain								0.619 **	0.612 **	0.368 *
TPC									0.736 **	0.617 **
TFC										0.724 **

Chl *a* = chlorophyll *a* (μg·g^−1^); chl *b* = chlorophyll *b* (μg·g^−1^); T Chl = total chlorophyll (μg·g^−1^); TCC = total carotene content (μg·g^−1^); TPC = total polyphenol content (µg/g GAE dw); TFC = total flavonoid content (µg/g QE dw); TAA = total antioxidant activity (%); * significant at 5% level, ** significant at 1% level, (*n* = 5).

## Data Availability

The data that are recorded in the current study are available in all of the tables and figures of the manuscript.
